# Fundamental limits in structured principal component analysis and how to reach them

**DOI:** 10.1073/pnas.2302028120

**Published:** 2023-07-18

**Authors:** Jean Barbier, Francesco Camilli, Marco Mondelli, Manuel Sáenz

**Affiliations:** ^a^Quantitative Life Sciences and Mathematics Sections, International Centre for Theoretical Physics, Trieste 34151, Italy; ^b^Institute of Science and Technology Austria, Klosterneuburg 3400, Austria; ^c^Centro de Matemática, Universidad de La República, Montevideo 11400, Uruguay

**Keywords:** high-dimensional inference, structured data, principal components analysis, replica method, approximate message passing

## Abstract

How do statistical dependencies in measurement noise influence high-dimensional inference? To answer this, we study the paradigmatic spiked matrix model of principal components analysis (PCA), where a rank-one matrix is corrupted by additive noise. We go beyond the usual independence assumption on the noise entries, by drawing the noise from a low-order polynomial orthogonal matrix ensemble. The resulting noise correlations make the setting relevant for applications but analytically challenging. We provide characterization of the Bayes optimal limits of inference in this model. If the spike is rotation invariant, we show that standard spectral PCA is optimal. However, for more general priors, both PCA and the existing approximate message-passing algorithm (AMP) fall short of achieving the information-theoretic limits, which we compute using the replica method from statistical physics. We thus propose an AMP, inspired by the theory of adaptive Thouless–Anderson–Palmer equations, which is empirically observed to saturate the conjectured theoretical limit. This AMP comes with a rigorous state evolution analysis tracking its performance. Although we focus on specific noise distributions, our methodology can be generalized to a wide class of trace matrix ensembles at the cost of more involved expressions. Finally, despite the seemingly strong assumption of rotation-invariant noise, our theory empirically predicts algorithmic performance on real data, pointing at strong universality properties.

## Significance

The assumption of unstructured “noise,” i.e., that the complement of what is considered an interesting “signal” in a certain dataset is pure randomness, has pervaded analytical studies in high-dimensional inference. However, this hypothesis is often too simplistic to capture realistic scenarios. We thus need to understand the role of the noise structure, namely the presence of correlations within it. We address this problem in spiked matrix models and provide characterization of the information-theoretic limits to inference. We also propose a message-passing algorithm which is observed through simulations to saturate these limits by optimally capturing the noise statistical dependencies. The resulting picture shows that both signal and noise structure should be exploited by algorithms in order to produce better signal estimation.

The success of inference and learning algorithms depends strongly on the structure of the high-dimensional noisy data they process. Consequently, quantifying how this structure helps algorithms to overcome the curse of dimensionality has become a central topic in statistics and machine learning. Classical examples include sparsity in compressed sensing (1), low-rank structure in matrix recovery (2), or community structure in community detection (3). In all these models, structure is usually assumed only at the signal’s level. But the decomposition of the data into “signal” (the component considered of interest) and “noise” (the rest) is often arbitrary and application dependent. For example, in the classification of “dogs/cats,” the training images contain a lot of information unrelated to dogs and cats—e.g., on the notions of “inside/outside,” “day/night,” etc. Yet, this highly structured potential source of information is discarded as random noise (independent, Gaussian, etc.). Most of the research effort has thus focused on understanding how the signal structure alone helps inferring it. In contrast, much less is known about the role of the noise structure and how to exploit it to improve inference.

Given their ubiquitous appearance in the statistics literature, spiked matrix models, which were originally formulated as models for probabilistic principal component analysis (PCA) (4), are now a paradigm in high-dimensional inference. Thanks to their universality features, they, and their generalizations, find numerous applications in other central problems, including community detection (3), group synchronization (5), and submatrix localization or high-dimensional clustering (6). They thus offer the perfect benchmark to quantify the influence of noise structure. In this paper, we focus on the following estimation problem: A statistician needs to extract a rank-one matrix (the spike) P^*^ := X^*^X^*⊺^, X^*^ ∈ ℝ^*N*^, from the data

[1] $$\begin{array}{c} Y=\frac{\lambda }{N}P^{*}+Z\in R^{N\times N}, \end{array}$$

with “noise” **Z** and signal-to-noise ratio (SNR) *λ* ≥ 0.

The spectral properties of finite rank perturbations of large random matrices like Eq. **1** were intensively investigated in random matrix theory (see, e.g., refs. 7–9), showing the presence of a threshold phenomenon coined BBP transition (in reference to the authors of ref. 7): When *λ* is large enough, the top eigenvalue of **Y** detaches from the bulk of eigenvalues. Its corresponding eigenvector has then a nontrivial projection onto the sought ground truth X^*^ and can be used as its estimator. The problem has also been approached from the angle of Bayesian inference (10–13). In particular, besides the previous spectral estimator, there exists a whole family of iterative algorithms, known as approximate message passing (AMP), that can be tailored to take further advantage of prior structural information about the signal and noise. AMP algorithms were first proposed for estimation in linear models (14, 15) but have since been applied to a range of statistical estimation problems, including generalized linear models (16, 17) and low-rank matrix estimation (11, 18). An attractive feature of AMP is that its performance in the high-dimensional limit can often be characterized by a succinct recursion called state evolution (19, 20). Using the state evolution analysis, it has been proved that AMP achieves Bayes-optimal performance for some models (11, 16, 18), and a conjecture posits that for a wide range of estimation problems, AMP is optimal among polynomial-time algorithms (21).

The references mentioned above rely on the assumption of independent and identically distributed (i.i.d.) noise, often taken Gaussian *Z*_*ij*_ = *Z*_*ji*_ ∼ 𝒩(0, 1), under which Eq. **1** is the well-known spiked Wigner model (4). This independence, or “absence of structure,” in the noise simplifies greatly the analysis. In order to relax this property, we may seek inspiration from the statistical physics literature on disordered systems. An idea that was first brought forth in refs. 22 and 23 for the Sherrington–Kirkpatrick model, and later imported also in high-dimensional inference (24, 25), is that of giving an inhomogeneous variance profile to the noise matrix elements; we mention that this idea in inference is similar to the earlier definition of “spatially coupled systems” (26, 27) in coding theory, see ref. 12 for its use in the present context. The procedure makes the (*Z*_*ij*_) no longer identically distributed, but it leaves them independent. This is an important step toward more structure in the noise. Yet, the independence assumption is a rather strong one. In fact, ref. 25 showed that a broad class of observation models, as long as the independence assumption holds, are information-theoretically equivalent to one with independent Gaussian noise.

One way to go beyond is to consider noises belonging to the wider class of rotationally invariant matrices. Since the appearance of the seminal studies (28–30), there has been a remarkable development in this direction, as evidenced by the rapidly growing number of papers on spin glasses (31–33) and inference (34–37) that take into account structured disorder, including the present one. Indeed, we hereby consider a spiked model in which the noise **Z** is drawn from an orthogonal matrix ensemble different from the Gaussian orthogonal ensemble (the only one with independent entries). Intuitively, the presence of dependencies in the noise should be an advantage for an algorithm sharp enough to see patterns within it and use them to retrieve the sought low-rank matrix. Going in that direction, Fan (35) proposed a version of AMP designed for rotationally invariant noises (using earlier ideas of refs. 31 and 32). Furthermore, in a recent work (38), part of the authors analyzed a Bayes estimator and an AMP, both assuming Gaussian noise, whereas the actual noise in the data was drawn from a generic orthogonal matrix ensemble. However, besides intuition and the mentioned studies, to the best of our knowledge, there is little theoretical understanding of the true role played by noise structure in spiked matrix estimation and more generically in inference. In particular, prior to our work, there was no theoretical prediction of optimal performance to benchmark practical inference algorithms.

## 1. Setting and Main Results

Our analysis focuses on two types of signal’s distributions: the factorized prior *dP*_*X*_(**x**)=∏_*i* ≤ *N*_*dP*_*X*_(*x*_*i*_) and a uniform prior measure over the *N*-dimensional sphere of radius $\sqrt{N}$. By convention, ∫*x*^2^ *dP*_*X*_(*x*)=1, which amounts to rescale *λ*. The noise matrix **Z** is drawn from a trace random matrix ensemble, defined by a certain potential *V* : ℝ ↦ ℝ. *V* is extended to matrices as follows: if **A** = diag(*a*_1_, …, *a*_*N*_) then *V*(**A**)=diag(*V*(*a*_1_),…,*V*(*a*_*N*_)). For real symmetric matrices **M** = **UAU**^⊺^, with **U** orthogonal, *V*(**M**)=**U***V*(**A**)**U**^⊺^. With these notations, we can write the density of the trace ensemble (with normalization constant *C*_*V*_) as

[2] $$\begin{array}{c} dP_{Z}(Z)=C_{V}\exp(-\frac{N}{2}\;\text{Tr}V(Z))\prod_{i\leq j}dZ_{ij}. \end{array}$$

Instances of such ensembles have a spectral decomposition **Z** = **ODO**^⊺^, with **O** uniformly distributed over *N* × *N* orthogonal matrices. The distribution of the eigenvalues in the diagonal matrix **D**, which is independent of **O**, can be explicitly written, see *SI Appendix*, section 1.2. Only the special case *V*(*x*)=*x*^2^/(2*σ*), corresponding to the Gaussian orthogonal ensemble, induces independent (Gaussian distributed) matrix entries. Any other potential generates dependencies among matrix elements and thus structure. For example, if we take *V*(*x*)=*x*^4^/4, the probability density would be proportional to $\prod \exp(-\frac{N}{8}Z_{\mathrm{ij}}Z_{\mathrm{jk}}Z_{\mathrm{kl}}Z_{\mathrm{li}})$, which is clearly not factorizable over matrix entries.

Analyzing the model for a generic potential *V* is possible through the methodology presented in this paper. Indeed, as discussed in *SI Appendix*, A, this can be done by studying the inference problem whose noise’s potential is a polynomial approximation of *V*. However, if we take a generic polynomial potential *V*, the higher the order, the more technical and cumbersome our derivations become. Therefore, for the sake of clarity, we focus on a concrete example of nontrivial correction to i.i.d. noise: the quartic matrix potential *V*(*x*)=*μx*^2^/2 + *γx*^4^/4, where *μ* and *γ* are two nonnegative real numbers (39). We could have also considered a nonsymmetric potential with a cubic term too, but for simplicity, we restrict ourselves to that case as symmetry slightly simplifies the computations. The noise Z drawn from the quartic matrix ensemble has a known *N* → ∞ asymptotic eigenvalue distribution (40)

[3] $$\begin{array}{c} \rho \left(x\right)dx=\left(\mu +2a^{2}\gamma +\gamma x^{2}\right)\sqrt{4a^{2}-x^{2}}/\left(2\pi \right)\;dx, \end{array}$$

where $a^{2}:=\left(\sqrt{\mu^{2}+12\gamma }-\mu \right)/\left(6\gamma \right)$. In order to have a coherent definition of SNR, we also fix ∫*x*^2^*dρ*(*x*)=1, which implies $\gamma =\gamma \left(\mu \right)=\left(8-9\mu +\sqrt{64-144\mu +108\mu^{2}-27\mu^{3}}\right)/27$. When *μ* = 1, *γ*(1)=0 and we recover the pure Wigner case. On the contrary, (*μ* = 0, *γ*(0)=16/27) corresponds to a purely quartic case with unit variance, the “most structured” ensemble in this class. Therefore, *μ* allows us to interpolate between unstructured and structured noise ensembles.

We emphasize that, although this model may seem rather academic at first sight, we will see that our main assumption, that is, the rotational invariance of the noise, turns out to yield a theory which accurately predicts the empirical performance of algorithms for inference of low-rank matrices hidden in noise coming from real datasets from various application domains. This is probably a consequence of strong universality properties, yet to be understood from a theoretical perspective, along the lines of refs. 41 and 42. We thus argue that our assumptions are in fact rather mild, making our inference algorithms relevant for potential future applications.

We now introduce the Bayesian framework we are going to analyze. Let **P** := **xx**^⊺^. The posterior measure reads

[4] $$\begin{array}{c} dP_{X|Y}\left(x|Y\right)\;=\;\frac{C_{V}}{P_{Y}\left(Y\right)}dP_{X}\left(x\right)\exp(\;-\frac{N}{2}\;\text{Tr}V(Y-\frac{\lambda }{N}P)\;). \end{array}$$

The evidence $P_{Y}\left(Y\right)$ is simply the integral of the numerator. We stress that the prior *P*_*X*_ and the likelihood *P*_*Y* ∣ *X*_ match respectively the distribution of the signal and the noise density *P*_*Z*_, and *λ* is known. Therefore, we are in the Bayes-optimal setting. Studying the limits of inference in this setting draws a fundamental line between what is information-theoretically possible and what is not in terms of performance of inference.

A main object of interest is the free entropy, which is minus the Shannon entropy of the data: $F_{N}\left(Y\right):=-H\left(Y\right)=E\ln P_{Y}\left(Y\right)$. It is related to the mutual information between signal and data through the identity $I\left(P^{*};Y\right)=-F_{N}\left(Y\right)+\ln C_{V}-\frac{N}{2}E\;\text{Tr}V\left(Z\right)$. The relevance of the latter is extensively discussed in *SI Appendix*, section 1.4. Using the form of the observation model in Eq. **1**, it reads

[5] $$\begin{array}{c} -I\left(P^{*};Y\right)=E\ln\int dP_{X}\left(x\right)e^{-H_{N}\left(x;Z,X^{*}\right)}=:E\ln Z, \end{array}$$

where the Hamiltonian linked to the partition function 𝒵 is

[6] $$\begin{array}{c} H_{N}\left(x;Z,X^{*}\right):=\frac{N}{2}\;\text{Tr}[V(Z+\frac{\lambda }{N}\left(P^{*}-P\right))-V\left(Z\right)]. \end{array}$$

In this way, the problem is mapped onto a statistical mechanics model with “quenched randomness” $Z,X^{*}$ and “spins” x with Gibbs–Boltzmann distribution associated with this Hamiltonian (i.e., the posterior). This Hamiltonian is tricky to directly deal with, so a key point will be to “convert” it into a more tractable quadratic form; see Section 1 and *SI Appendix*, section 3.1.

### Result 1: Information-Theoretical Limits.

Our first result is a variational formula for the mutual information via the celebrated replica method (43) outlined in Section 1: If we let τ_*_ := argmax{*f*_*ρ*_(**τ**):**τ** ∈ ℝ^13^, ∇*f*_*ρ*_(**τ**)=***0***}, then we have the following low-dimensional expression for the mutual information between hidden spike and the data:

[7] $$\begin{array}{c} \frac{1}{N}I\left(P^{*};Y\right)\overset{N\to \infty }{\to }-\;f_{\rho }\left(\tau_{*}\right). \end{array}$$

The argmax is selected and not the argmin as *f*_*ρ*_ is a free entropy (i.e., minus free energy, the free energy being minimized in physics). *f*_*ρ*_ and its derivation are reported in *SI Appendix*, section 3.2. The 13 coupled fixed point equations coming from ∇*f*_*ρ*_ = ***0*** will reduce to only 2 (*SI Appendix*, Eqs. **79**–**84**) thanks to special symmetries inherent to the Bayes-optimal nature of our analysis. One of the two remaining order parameters, denoted *m*^2^ and called (squared) “magnetization,” quantifies the asymptotic trace inner product between the minimum mean-square error (MMSE) estimator $\int dP_{X|Y}\left(x|Y\right)xx^{⊺}$ and the spike $X^{*}X^{*⊺}$. It allows us to compute the MMSE as

[8] $$\begin{array}{c} \frac{1}{2N^{2}}E{\left\|X^{*}X^{*⊺}-\int dP_{X|Y}\left(x|Y\right)xx^{⊺}\right\|}_{\;\text{F}}^{2}\overset{N\to \infty }{\to }\frac{1-m^{2}}{2}, \end{array}$$

with *m* solving the aforementioned system of equations.

### Result 2: Optimality of PCA for Rotationally Invariant Priors.

The above results hold for a factorized prior *P*_*X*_^⊗*N*^. Nevertheless, if $X^{*}$ is uniformly distributed on the sphere, a variational formula analogous to Eq. **7** can still be derived, as shown in *SI Appendix*, section 3.6, and the related MMSE computed. Analytical arguments and numerical experiments show that the latter can be achieved using the naive spectral estimator $C\nu \nu^{⊺}$ of P^*^ obtained from the principal eigenvector ν=ν(Y) of Y properly rescaled by a certain factor *C*(*λ*, *ρ*); see ref. 9.

### Result 3a: Optimal Preprocessing of the Data.

Instead of using an AMP with iterates based on **Y**, we introduce a preprocessing procedure driven by the AdaTAP formalism (32). The end result is an effective quadratic model (i.e., with only pairwise interactions) which is “equivalent” (in a proper sense described below) to the original one, with coupling matrix

[9] $$\begin{array}{c} J\left(Y\right)=\mu \lambda Y-\gamma \lambda^{2}Y^{2}+\gamma \lambda Y^{3}. \end{array}$$

This model being quadratic is now solvable using AdaTAP/AMP and possesses the same thermodynamic properties (free entropy, phase transitions, etc.) as well as the same marginal means and variances as the model in Eq. **4** when *N* → ∞ (and thus equivalent for our purposes). Therefore, to approximate the MMSE estimator, one can simply “preprocess” Y by applying J(Y) and then efficiently compute the marginals of the resulting quadratic model by AdaTAP/AMP, see next section. AdaTAP allows us to parametrize the free entropy (i.e., log-partition function) of a model with quadratic Hamiltonian, for a given instance of the interaction matrix, in terms of *O*(*N*) order parameters, some of which correspond to the sought marginal means (⟨*x*_*i*_⟩)_*i* ≤ *N*_ and associated variances. The extremization w.r.t. them yields equations that can be solved iteratively and identified with an AMP algorithm. However, the Hamiltonian in Eq. **6** is not quadratic in **x** but can be made so by fixing certain order parameters as outlined in Section 1. The resulting coupling matrix depends on **Y** and on the fixed order parameters, whose values are constrained by Bayes optimality (*SI Appendix*, section 1.4). Using these values, for an initial quartic *V*(*x*)=*μx*^2^/2 + *γx*^4^/4, we get the above interaction matrix in Eq. **9** (*SI Appendix*, sections 5.1 and 5.2).

**Fig. 1.**
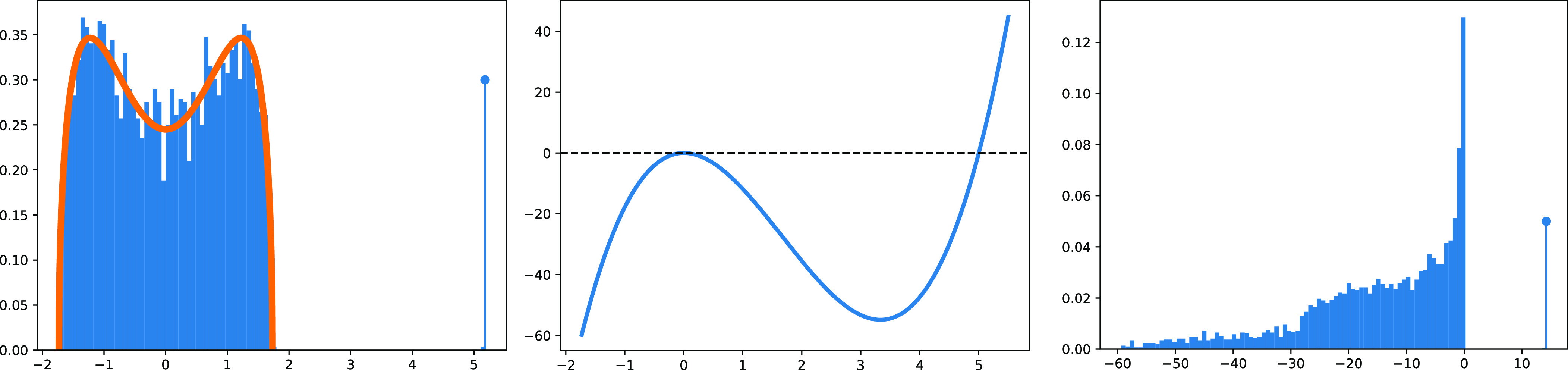
We have set *μ* = 0, *γ*(0)=16/27, *λ* = 5, *N* = 4,000 and generated one instance of the data model Eq. **1**. (*Left*) Histogram of the eigenvalues of **Y**. The leading eigenvalue is emphasized, and the orange curve is the density in Eq. **3**. (*Middle*) Optimal preprocessing function *J*(*x*)=*μλx* − *γλ*^2^*x*^2^ + *γλx*^3^. (*Right*) Histogram of the eigenvalues of J(Y). The preprocessing *J* flushes the bulk to the negative axis while pushing only the leading eigenvalue even further from the bulk in the positive direction.

The “cleaning effect” of J(Y) is illustrated in Fig. 1. In general, for a (*K* + 1)-order polynomial matrix potential, the preprocessed matrix is a polynomial $J\left(Y\right)=\sum_{k\leq K}c_{k}Y^{k}$, with (*c*_*k*_)_*k* ≤ *K*_ depending on *V*. For example, for *V*(*x*)=*ξx*^6^/6 (with *ξ* = 27/80 to select unit variance), the preprocessing (derived similarly to the quartic case, see *SI Appendix*, section 5.3) is *J*_6_(*x*)=*ξλx*^5^ − *ξλ*^2^*x*^4^ − *ξλ*^2^*x*^2^; it has an effect similar to that in Fig. 1. We point out that the statistics of the noise could be only partially known. This issue can be overcome by learning the (*c*_*k*_) from the data; see *SI Appendix*, B.

### Result 3b: Bayes-Optimal AMP.

First, we show in *SI Appendix*, section 4 that existing AMPs (35, 36) do not saturate the MMSE predicted by Eq. **8**. We provide a replica-based theory showing that despite these existing AMPs are aware of the noise structure/statistics, they nevertheless make an implicit mismatched assumption of i.i.d. Gaussian noise: The noise structure is “only” exploited to enforce convergence despite the mismatch, rather than as a source of greater statistical accuracy, in contrast to the proposed AMP we explain now.

To cure this issue, we employ the preprocessed J(Y) in AMP, which leads to our (conjectural) Bayes-optimal approximate message-passing (BAMP) algorithm with recursion

[10] $$\begin{array}{c} f^{t}=J\left(Y\right)u^{t}-\sum_{i\leq t}c_{t,i}u^{i},\;u^{t+1}=g_{t+1}\left(f^{t}\right),\;t\geq 1, \end{array}$$

with *g*_*t* + 1_ applied component-wise. For simplicity, we assume to have access to an initialization $u^{1}\in R^{N}$ independent of the noise Z and with a strictly positive correlation with $X^{*}$, i.e.,

[11] $$\begin{array}{c} \left(X^{*},u^{1}\right)\overset{W_{2}}{⟶}\left(X^{*},U_{1}\right),\;\;E\left[X^{*}\;U_{1}\right]:=\epsilon >0,\;\;E\left[U_{1}^{2}\right]=1. \end{array}$$

This requirement is rather standard in the analysis of AMP algorithms (16, 35, 44). However, as having access to such an initialization is often impractical, recent work (18, 36, 45) has designed AMPs initialized with the top eigenvector ν(Y).

By carefully choosing the Onsager coefficients {c_*t*, *j*_}_*j* ∈ [*t*]_, we rigorously obtain BAMP’s state evolution characterization.

Theorem 1 (State evolution of BAMP). Let $J\left(Y\right)=\sum_{i\leq K}c_{i}Y^{i}$. Consider the AMP of Eq. **10** initialized as Eq. **11**, with Onsager coefficients {c_*t*, *j*_}_*j* ∈ [*t*]_ given in *SI Appendix*, section 6.2, and where (*g*_*t* + 1_)_*t* ≥ 1_ are 𝒞^1^ and Lipschitz. Then, the following limit holds almost surely for any order 2 pseudo-Lipschitz function^*^$\psi :R^{2t+2}\to R$ and *t* ≥ 1:

[12] $$\begin{array}{cc} & \frac{1}{N}\sum_{i\leq N}\psi \left(u_{i}^{1},\ldots ,u_{i}^{t+1},f_{i}^{1},\ldots ,f_{i}^{t},X_{i}^{*}\right)\\ & \;\;\overset{N\to \infty }{\to }E\;\psi \left(U_{1},\ldots ,U_{t+1},F_{1},\ldots ,F_{t},X^{*}\right). \end{array}$$

Here, for *i* ∈ [*t*], *U*_*i* + 1_ = *g*_*i* + 1_(*F*_*t*_) and (*F*_1_, …, *F*_*t*_)=***μ***_*t*_X^*^ + (*W*_1_, …, *W*_*t*_), with (*W*_*i*_)_*i* ≤ *t*_ a multivariate Gaussian vector whose covariance as well as ***μ***_*t*_ are given in *SI Appendix*, section 6.2.

Eq. **12** provides a high-dimensional characterization of our proposed BAMP. A suitable choice of *ψ* readily gives the MSE of the BAMP iterates. We also note that our result is equivalent to the almost sure convergence in Wasserstein-2 distance of the joint empirical distribution of $\left(u^{1},\ldots ,u^{t+1},f^{1},\ldots ,f^{t},X^{*}\right)$ to (*U*_1_, …, *U*_*t* + 1_, *F*_1_, …, *F*_*t*_, X^*^), see corollary 7.21 of ref. 44.

We emphasize that our BAMP algorithm is not the usual AMP of ref. 35, where the data matrix Y are just replaced by the preprocessed matrix J(Y). Indeed, tuning the Onsager coefficients {c_*t*, *i*_} entering BAMP requires a type of “multistage” state evolution recursion which is completely different from the one in ref. 35. The acronym we introduce stresses this crucial distinction. While our replica prediction for the MMSE is nonrigorous, the state evolution analysis of BAMP is rigorous. In Section 2, we show that BAMP improves over the AMP in ref. 35 by comparing their fixed points. This improvement is thus a rigorous conclusion, while the conjecture is that BAMP saturates the Bayes-optimal performance.

Finally, the “multistage” state evolution of BAMP suggests a choice of the denoisers in the AMP of ref. 35, which differs from the greedy strategy of ref. 36 (i.e., picking the full posterior mean denoiser at every iteration). The numerical results of Section 2 also show that this denoiser selection—motivated by BAMP—meets the BAMP performance and, hence, the replica prediction of the Bayes-optimal error.

## 2. Numerical Results and Discussion

### BAMP vs the Replica Prediction.

**Fig. 2.**
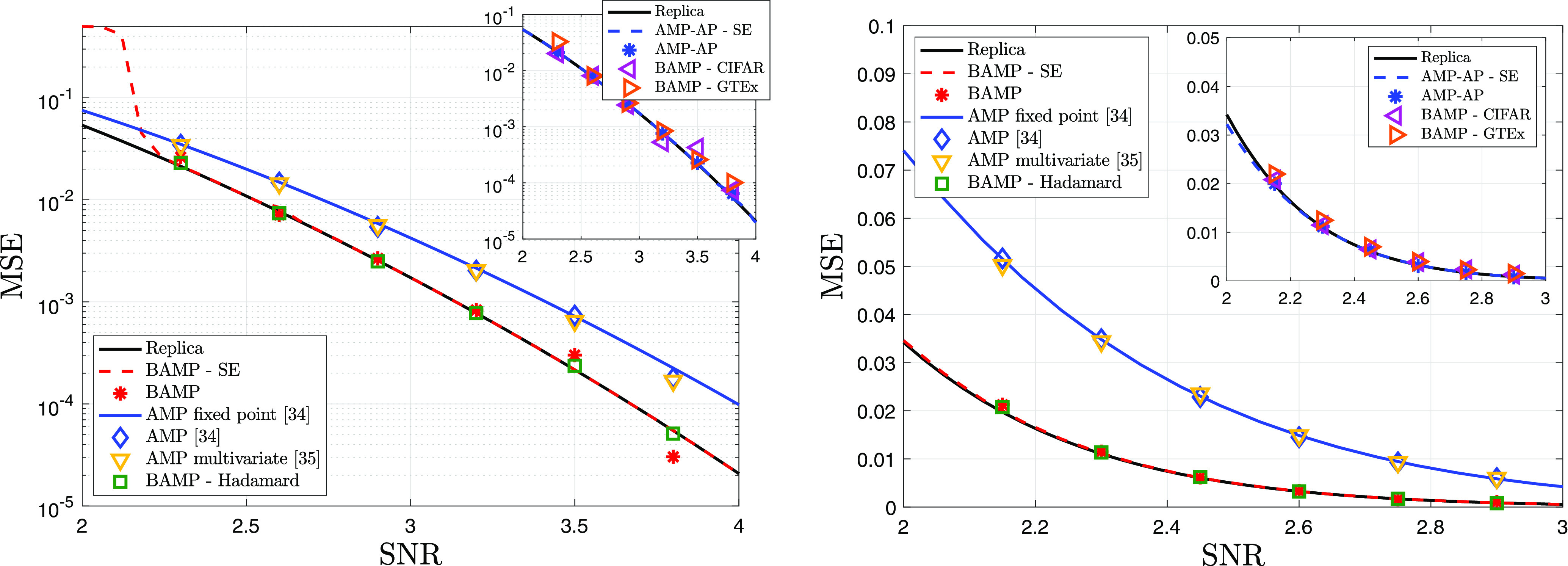
Quartic potential with *μ* = 0 (*Left*) and pure power six potential (*Right*). Comparison of the following inference procedures: (*i*) (black) replica prediction of the MMSE, Eq. **8**. (*ii*) (red) Performance of the BAMP algorithm, where *g*_*t* + 1_ is the single-iterate posterior mean denoiser *g*_*t* + 1_(*f*)=𝔼[X^*^ ∣ *F*_*t*_ = *f*]. The red line corresponds to the fixed point of the MSE given by the state evolution recursion, and the red stars denote the MSE obtained by running BAMP Eq. **10** with the proper preprocessing. (*iii*) (blue) Performance of the AMP proposed in ref. 35. The blue line corresponds to the fixed point of the MSE obtained with a single-iterate posterior mean as denoiser, and the blue diamonds denote the MSE obtained by running the AMP of ref. 35 with the same denoiser. (*iv*) (ochre squares) MSE obtained by the AMP of ref. 36 (without the preprocessing of **Y**), which employs a full memory posterior mean denoiser: $h_{t+1}\left(f_{1},\ldots ,f_{t}\right)=E\left[X^{*}|\left(F_{1},\ldots ,F_{t}\right)=\left(f_{1},\ldots ,f_{t}\right)\right]$. Finally, (*v*) (green triangles) performance of BAMP when the uniformly distributed matrix **O** (appearing in the spectral decomposition of the noise Z) is replaced by the product of the Hadamard–Walsh matrix and a diagonal matrix with i.i.d. Rademacher entries as in ref. 42. In the smaller plots in the *Top-Right* corner, we report the performance of AMP-AP (blue) and of BAMP for our universality experiments involving the CIFAR-10 “plane” class (purple) and the “muscle skeletal” GTEx dataset (orange).

The *Left* plot of Fig. 2 considers the quartic ensemble for *μ* = 0, and the right one refers to the pure power six potential. The signal $X^{*}$ has a Rademacher prior $X_{i}^{*}∼\frac{1}{2}\left(\delta_{1}+\delta_{-1}\right)$. The estimators of the spike $X^{*}X^{*⊺}$ are compared in terms of the MSE achieved at the fixed point, as a function of the SNR *λ*. All algorithms are run for *N* = 8,000, they are initialized with **u**^1^ that satisfies Eq. **11**, and the results are averaged over 50 trials; the state evolution recursions and the replica prediction are for *N* → ∞. In *SI Appendix*, section 7.2, we provide additional numerical results for a sparse Rademacher prior, which display a similar qualitative behavior.

We observe that all algorithms converge rapidly: 10 iterations are sufficient to reach the corresponding fixed points. A few remarks concerning the results displayed in Fig. 2 are now in order. First, in all settings, the fixed point of the BAMP state evolution (red) matches the replica prediction (black). This is a strong numerical evidence supporting our conjecture that the proposed BAMP algorithm is Bayes-optimal. These theoretical curves for *N* → ∞ are also remarkably close to the MSE achieved by the BAMP algorithm at *N*= 8,000.

Second, there is a clear performance gap between our proposed BAMP (red) and the existing AMP algorithms (35, 36) (single-step denoiser in blue, and multistep in ochre). For *V*(*x*)=*ξx*^6^/6, the gap is even more evident. As predicted by our theory, the gap is reduced when *μ* approaches 1 with all curves collapsing for *μ* = 1; see *SI Appendix*, section 7.2.

Thirdly, we consider a choice of denoisers in the AMP of ref. 35, which is motivated by our BAMP: If the potential has degree *K*, every *K*-th nonlinearity is the full memory posterior mean denoiser, and all the other denoisers are chosen to be the identity. The algorithm is dubbed AMP with alternating posteriors (AMP-AP), and its connection to BAMP is discussed at the end of the 1 section. As evident from the smaller plots in the top right corner, AMP-AP (blue) matches the performance of BAMP and of the replica prediction as well.

Last, BAMP is numerically unstable for low SNR. For the quartic potential and *λ* = 2.3, 5 out of 50 trials do not reach the state evolution fixed point (and are thus discarded). Furthermore, BAMP’s state evolution detaches from the replica prediction as the SNR gets smaller. Considering an initialization closer to the fixed point mitigates the issue. This instability is likely due to the fact that BAMP’s state evolution corresponds to an auxiliary AMP that multiplies the number of iterations (see the 1 section) and which thus amplify errors.

### Universality of the Rotational Invariance Assumption.

We believe that our results apply beyond the rotational invariance assumption to cases where the eigenbasis of the noise is invariant under more restrictive transformations (such as permutations), or even “quasideterministic.” This intuition comes from recent studies (41, 42) showing that, when AMP or its linearized version are used, the class of rotationally invariant matrices leads to the same performance as a much broader class of matrices (with same spectral density). While the existing literature considers a setting different than ours, this still suggests that our predictions should remain true more generally. To confirm this, we plot in Fig. 2 the performance of BAMP when the uniformly distributed matrix **O** (i.e., the noise Z eigenbasis) is replaced by i) the product of the Hadamard–Walsh matrix and a diagonal matrix with i.i.d. Rademacher entries, as in ref. 42 (green squares), or ii) the eigenbasis of the covariance matrix for two popular datasets in computer vision and quantitative genetics, i.e., the CIFAR-10 (46) “plane” class and the “muscle skeletal” GTEx dataset (47, 48) (purple and orange markers, respectively, in the *Top-Right* plots). The excellent match clearly supports the universality of our predictions. Additional validations are contained in *SI Appendix*, section 7.2. These results can be understood from the fact that any eigenbasis **O** is typical w.r.t. the Haar measure, so for a fixed instance, as long as **O** is sufficiently independent of the eigenvalues, the universality should hold. This suggests that, in practice, our rotational invariance assumption effectively corresponds to assuming decoupling between eigenbasis and eigenvectors.

## 3. Methods

### Outline of the Replica Computation.

The starting point of the replica method is the “replica trick” lim_*N* → ∞_𝔼ln𝒵(**Y**)/*N* = lim_*n* → 0_lim_*N* → ∞_ln𝔼𝒵^*n*^(**Y**)/(*Nn*) that implicitly assumes the commutation of the *n*, *N* limits. Another key assumption is to consider *n* ∈ ℕ in the computation and then assume an analytic continuation to *n* close to 0_+_. The expectation is with respect to Y or equivalently the independent $O,X^{*}$; concerning **D**, we only need that its empirical eigenvalue distribution converges weakly to *ρ* and that it has asymptotically no outliers. When computing 𝒵^*n*^, we get multiple integrals over (**x**_ℓ_)_0 ≤ ℓ ≤ *n*_, with **x**_0_ ≡ X^*^, and a sum of *n* Hamiltonians as in Eq. **6** in the exponential. Expanding the exponent, we identify some order parameters: For 1 ≤ ℓ ≤ *n*,

$$\begin{array}{cc} & v_{\ell }:=\frac{\|x_{\ell }{\|}^{2}}{N},\;\;M_{(k)\ell }:=\frac{x_{\ell }^{⊺}Z^{k}x_{\ell }}{N},\;\;\kappa_{\ell }:=\frac{x_{\ell }^{⊺}Zx_{0}}{N},\;\;m_{\ell }:=\frac{x_{0}^{⊺}x_{\ell }}{N}, \end{array}$$

After fixing these using the Fourier representation of the Dirac delta function, the replicated partition function reads

$$\begin{array}{c} EZ^{n}=E_{Z,x_{0}}\int \prod_{l=1}^{n}dP_{X}(x_{l})d\tau_{l}d{\hat{\tau }}_{l}e^{-H_{N}(\tau_{l},{\hat{\tau }}_{l},x_{l};x_{0},Z)}, \end{array}$$

where $H_{N}\left(\tau ,\hat{\tau },x;x_{0},Z\right):=Nh\left(\tau ,\hat{\tau }\right)+x^{⊺}J_{1}\left(\tau ,\hat{\tau },Z\right)x+x^{⊺}J_{0}\left(\tau ,\hat{\tau },Z\right)x_{0}$, and **τ**_ℓ_ := (*v*_ℓ_, *M*_(1)ℓ_, *κ*_ℓ_, *m*_ℓ_) with ${\hat{\tau }}_{\ell }$ being the Fourier conjugate. The definitions of (*h*, **J**_1_, **J**_0_) can be found in *SI Appendix*, section 3.1. This point is crucial as it allows us to write the *n* Hamiltonians (one per **x**_ℓ_) as at most quadratic functions of **x**_ℓ_. Due to the quartic nature of the potential, the original *H*_*N*_ would instead have quartic interactions, or higher-order ones for polynomial *V* of degree greater than four. Yet, by identifying the proper order parameters, a similar reduction to effective quadratic Hamiltonians would still be possible.

In 𝔼𝒵^*n*^, the replicas are coupled in the system only through the expectation over the quenched noise, that can be rewritten as an expectation over the Haar distributed noise eigenbasis 𝔼_**O**_. The entire computation then boils down to the evaluation of an inhomogeneous log-spherical integral that we introduced and defined as follows: Let the matrices **C**_ℓℓ′_ = diag((*C*_*i*, ℓℓ′_)_*i* ≤ *N*_), **C**_*i*_ = (*C*_*i*, ℓℓ′_)_ℓ, ℓ′≤*n*_, and vectors **h**_ℓ_ = (*h*_*i*, ℓ_)_*i* ≤ *N*_, **h**_*i*_ = (*h*_*i*, ℓ_)_ℓ ≤ *n*_ all having bounded entries uniformly in *N*. The sequence (**h**_*i*_ ∈ ℝ^*n*^, **C**_*i*_ ∈ ℝ^*n* × *n*^)_*i* ≤ *N*_ is assumed to have an empirical law tending to that of the random variable (**h** ∈ ℝ^*n*^, **C** ∈ ℝ^*n* × *n*^). The inhomogeneous log-spherical integral is defined as

$$\begin{array}{c} I_{N}\;:=\;\frac{1}{N}\ln E_{O}\exp\;(\sum_{\ell ,\ell^{\prime }\leq n}{\left(Ox_{\ell }\right)}^{⊺}C_{\ell \ell^{\prime }}Ox_{\ell^{\prime }}\;+\;\sum_{\ell \leq n}{\left(Ox_{\ell }\right)}^{⊺}h_{\ell }). \end{array}$$

Its limit depends only on the law of (**C**, **h**) and on the overlaps $q_{\ell \ell \prime }:=\frac{1}{N}x_{\ell }^{⊺}x_{\ell \prime }$, ℓ ≤ ℓ′, that we need to fix with additional Dirac deltas in addition to the previous order parameters. We find that lim_*N* → ∞_ℐ_*N*_ is expressed by a variational formula; see *SI Appendix*, section 2.1. This integral is a natural generalization of the standard spherical integral (49) and thus may have an interest beyond the present model, in particular, in random matrix theory or spin glasses.

The final ingredient is a replica symmetric ansatz, justified by the strong concentration-of-measure effects taking place in the Bayes-optimal setting (50, 51). It amounts to assume that all order parameters entering the model are independent of the replica index ℓ. Finally, a saddle point yields an extremization over ℝ^13^ of an effective action. Eqs. **7** and **8** follow directly.

Concerning the reduction from 13 to 2 order parameters (saddle point equations): This is possible thanks to a symmetry arising as a consequence of the Bayes rule which is specific to the Bayes-optimal setting and often called Nishimori identity. It allows to “interchange” the ground-truth signal $X^{*}$ with a sample x from the posterior Eq. **4** inside joint expectations over the posterior and data (see, e.g., ref. 51) and as a consequence to automatically fix the value of most order parameters.

### Auxiliary AMP and Onsager Coefficients.

The Onsager coefficients {c_*t*, *i*_}_*i* ∈ [*t*],*t* ≥ 1_ are designed so that, conditioned on the signal, the empirical distribution of the iterate **f**^*t*^ is Gaussian, namely $\left(f^{1},\ldots ,f^{t}\right)\overset{W_{2}}{⟶}\left(F_{1},\ldots ,F_{t}\right):=\mu_{t}X^{*}+W_{t}$, with $W_{t}∼N\left(0,\Sigma_{t}\right)$ for some mean vector ***μ***_*t*_ and covariance matrix ***Σ***_*t*_. For the AMP in ref. 35, this condition is enforced via the reduction to an *auxiliary* AMP, which also allows to track the iterates of the original algorithm and yields the state evolution parameters, such as ***μ***_*t*_ and ***Σ***_*t*_ above. This reduction crucially relies on splitting the matrix **Y** that multiplies the iterate **u**^*t*^, into the rank-one signal plus the noise matrix. In contrast, in Eq. **10**, the iterate is multiplied by the preprocessed matrix J(Y), which cannot be directly split in a similar fashion. Hence, we track all the contributions (**Y**^*k*^**u**^*t*^)_*k* ≤ *K*_, so that we can split them as $Y^{k}u^{t}=Y\;Y^{k-1}u^{t}=\frac{\lambda }{N}\;X^{*}\left\langle X^{*},Y^{k-1}u^{t}\right\rangle +ZY^{k-1}u^{t}$.

The key idea is to map the first *T* iterations of Eq. **10** to the first *K* × *T* iterations of an auxiliary AMP with iterates ${\left({\tilde{z}}^{t},{\tilde{u}}^{t}\right)}_{t\in [KT]}$ and denoisers ${\left\{{\tilde{h}}_{t+1}\right\}}_{t\in [KT]}$,

[13] $$\begin{array}{c} {\tilde{z}}^{t}=Z{\tilde{u}}^{t}-\sum_{i\leq t}{\overset{¯}{b}}_{t,i}{\tilde{u}}^{i},\;{\tilde{u}}^{t+1}={\tilde{h}}_{t+1}\left({\tilde{z}}^{1},\ldots ,{\tilde{z}}^{t},u^{1},X^{*}\right), \end{array}$$

whose state evolution can instead be deduced from ref. 35. The denoisers ${\left\{{\tilde{h}}_{t+1}\right\}}_{t\in [KT]}$ of this multistage auxiliary AMP are chosen so that, for *t* ∈ [*T*] and ℓ ∈ [*K*],

[14] $$\begin{array}{c} \frac{1}{N}{\left\|{\tilde{u}}^{K(t-1)+\ell }-Y^{\ell -1}u^{t}\right\|}_{2}^{2}\overset{N\to \infty }{\to }0. \end{array}$$

More specifically, for *t* ∈ [*T*] and ℓ ∈ {2, …, *K*}, the denoiser ${\tilde{h}}_{K(t-1)+\ell }$ giving ${\tilde{u}}^{K(t-1)+\ell }$ is a linear combination of past iterates ${\tilde{u}}^{1},\ldots ,{\tilde{u}}^{K(t-1)+\ell -1}$ and of ${\tilde{z}}^{K(t-1)+\ell -1}$; furthermore, the coefficients of these linear combinations are chosen to ensure that ${\tilde{u}}^{K(t-1)+\ell }\approx Y^{\ell -1}u^{t}$. Hence, by using Eq. **13** with *Kt* in place of *t*, one gets ${\left(Y^{\ell }u^{t}\right)}_{\ell \in [K]}$ from ${\tilde{z}}^{\mathrm{Kt}}$ and ${\left({\tilde{u}}^{K(t-1)+\ell }\right)}_{\ell \in \{2,\ldots ,K\}}$ (up to an *o*_*N*_(1)). Thus, $J\left(Y\right)u^{t}$ can be expressed as a linear combination of $\left({\tilde{u}}^{1},\ldots ,{\tilde{u}}^{\mathrm{Kt}},{\tilde{z}}^{\mathrm{Kt}}\right)$, which in turn is a linear combination of i) the past iterates {**u**^*i*^}_*i* ∈ [*t*]_, ii) the signal $X^{*}$, plus iii) independent Gaussian noise. By inspecting the coefficients of this linear combination, one deduces a) the Onsager coefficients {c_*t*, *i*_}_*i* ∈ [*t*],*t* ≥ 1_ (as the coefficients multiplying the past iterates {**u**^*i*^}_*i* ∈ [*t*]_), b) the mean *μ*_*t*_ (as the coefficient multiplying the signal $X^{*}$), and c) the covariance matrix ***Σ***_*t*_ (as the covariance matrix of the remaining noise terms). Finally, by making ${\tilde{h}}_{Kt+1}$ depend on *g*_*t* + 1_, we enforce that ${\tilde{u}}^{Kt+1}\approx u^{t+1}$. The description of the auxiliary AMP is deferred to *SI Appendix*, C.1, and its state evolution follows in *SI Appendix*, C.2.

In summary, the derivation of BAMP’s Onsager coefficients involves approximating ${\left\{Y^{k}u^{t}\right\}}_{k\leq K-1}$. This suggests an alternative choice of denoisers leading to the algorithm dubbed AMP-AP: For each batch of *K* iterations, we pick linear denoisers in the first *K* − 1 of them, as this allows to construct ${\left\{Y^{k}u^{t}\right\}}_{k\leq K-1}$; then, at the *K*-th iteration, we pick the posterior mean using all the past iterates, as this—in principle—allows one to assemble the vectors ${\left\{Y^{k}u^{t}\right\}}_{k\leq K-1}$ to obtain $J\left(Y\right)u^{t}$ as in BAMP. We note that AMP-AP does not require the coefficients of the polynomial J(Y), but it rather leaves to the posterior mean denoiser to learn them from the data. As such, it provides an efficient alternative to our proposed BAMP.

## Supplementary Material

Appendix 01 (PDF)

## Data Availability

All codes for generating the figures and to test the proposed AMP algorithm have been deposited in GitHub (https://github.com/fcamilli95/Structured-PCA-) (46). The CIFAR-10 dataset is available at (https://www.cs.toronto.edu/~kriz/cifar.html) (48). The GTEx dataset is available at (https://gtexportal.org) (52).

## Footnotes

^*^A function $\psi :R^{m}\to R$ is pseudo-Lipschitz of order 2 if there exists a constant *C* > 0 such that, for all $x,y\in R^{m}$, ${\left\|\psi \left(x\right)-\psi \left(y\right)\right\|}_{2}\leq {C(1+\|x\|}_{2}+{\left\|y\right\|}_{2}{)\|x-y\|}_{2}$.
